# Emergency Department Presentations to the Mental Health Services at Sligo University Hospital during the COVID pandemic

**DOI:** 10.1192/j.eurpsy.2024.335

**Published:** 2024-08-27

**Authors:** S. M. Gunawardena, A. Godavarthi, E. Gethins

**Affiliations:** ^1^Department of Liaison Psychiatry, Sligo University Hospital, Sligo, Ireland

## Abstract

**Introduction:**

In March 2020, the WHO declared the outbreak of SARS-CoV-2 a pandemic and Ireland went into its first lockdown. The Mental Health Liaison team at Sligo University Hospital operate 8 am to 8 pm, 7 days a week, with out of hours covered by the on-call Psychiatry junior doctors. The service has seen an increase in referrals, many of whom are known to community mental health teams.

**Objectives:**

We reviewed the numbers and trends of mental health presentations to the Emergency Department at Sligo University Hospital throughout the pandemic, including the patterns of presentations around the implementation of lockdowns.

**Methods:**

The Liaison Mental Health Service at Sligo University Hospital gathers data relating to numbers and types of presentation to the service. Data was taken from a pre-existing database of psychiatric presentations to the emergency department at Sligo University Hospital including patient demographics, nature of presenting complaint, time period in which they presented and whether they were previously linked in with a community mental health team in the preceding six months. We also looked at the pattern of ED mental health presentations from March 2019 to August 2021. In order to compare psychiatric presentations pre and during covid, data was broken down into two groups: the twelve months preceding March 2020 and the twelve months from March 2020. Categorical data were analysed using the Chi squared test for homogeneity in SPSS.

**Results:**

Overall, there was a 14% increase in mental health presentations during the pandemic. There was a significantly greater proportion of presentations of psychosis during the pandemic period (p<.014) and for medication review (p=.03) and significantly less presenting with addiction (p <0.001). Of those patients seen in the Emergency Department in 2021, 54.3% were known to the CMHT in the previous 6 months. in 2019, mental health presentations made up 1.36% of total ED attendances. This increased to 1.47% in 2020. From January to August 2021, 2.62% of ED attendances were mental health presentations.

**Image:**

**Figure:**
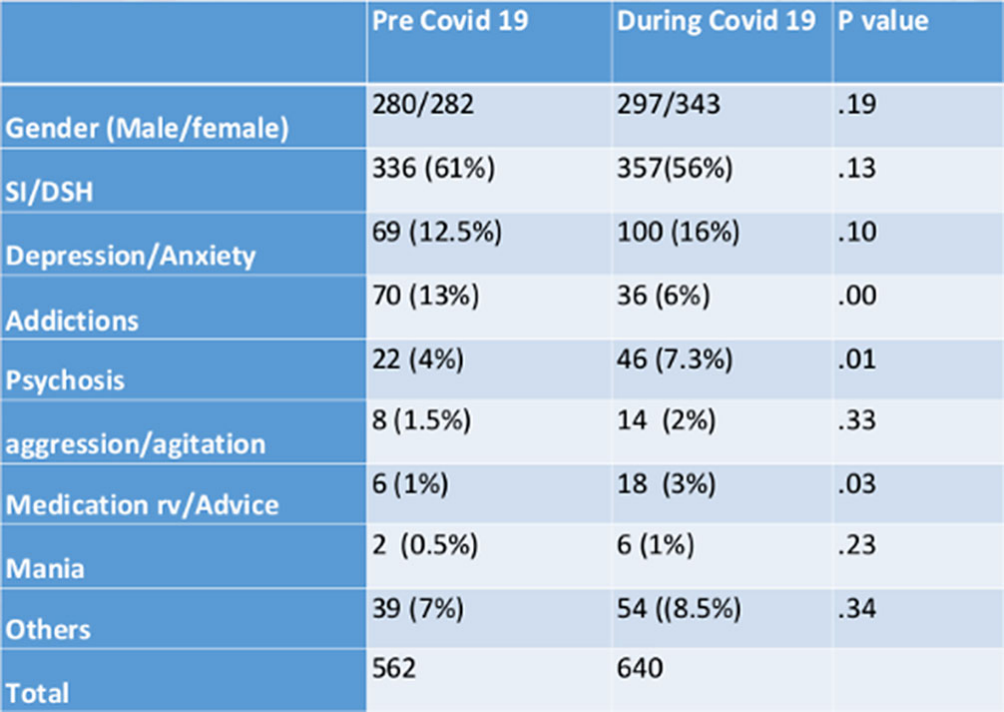

**Conclusions:**

The Covid-19 pandemic led to increased mental health presentations to Sligo University Hospital emergency department, with a significantly greater proportion presenting with psychosis and for medication review. These findings have implications around patient care and service provision. These results show that the prevalence of mental health has increased during the pandemic, particularly severe and enduring mental illnesses. New strategies must be implemented to accommodate to this increase in presentations.

**Disclosure of Interest:**

None Declared

